# Therapeutic Potential of the Molecular Chaperone and Matrix Metalloproteinase Inhibitor Clusterin for Dry Eye

**DOI:** 10.3390/ijms22010116

**Published:** 2020-12-24

**Authors:** M. Elizabeth Fini, Shinwu Jeong, Mark R. Wilson

**Affiliations:** 1 New England Eye Center, Tufts Medical Center and Department of Ophthalmology, Tufts University School of Medicine, Program in Pharmacology & Drug Development, Graduate School of Biomedical Sciences Tufts University, Boston, MA 02111, USA; 2 Department of Ophthalmology, USC Roski Eye Institute, Keck School of Medicine of USC, University of Southern California, Los Angeles, CA 90089, USA; shinwuje@med.usc.edu; 3 The Illawarra Health and Medical Research Institute, Molecular Horizons and the School of Chemistry and Molecular Bioscience, University of Wollongong, Wollongong, NSW 2522, Australia; mrw@uow.edu.au

**Keywords:** dry eye, ocular surface disease, corneal barrier function, animal models of dry eye, biomarkers, inflammation, molecular chaperone, matrix metalloproteinase

## Abstract

Evidence is presented herein supporting the potential of the natural homeostatic glycoprotein CLU (clusterin) as a novel therapeutic for the treatment of dry eye. This idea began with the demonstration that matrix metalloproteinase MMP9 is required for damage to the ocular surface in mouse dry eye. Damage was characterized by degradation of OCLN (occludin), a known substrate of MMP9 and a key component of the paracellular barrier. Following up on this finding, a yeast two-hybrid screen was conducted using MMP9 as the bait to identify other proteins involved. CLU emerged as a strong interacting protein that inhibits the enzymatic activity of MMP9. Previously characterized as a molecular chaperone, CLU is expressed prominently by epithelia at fluid-tissue interfaces and secreted into bodily fluids, where it protects cells and tissues against damaging stress. It was demonstrated that CLU also protects the ocular surface in mouse dry eye when applied topically to replace the natural protein depleted from the dysfunctional tears. CLU is similarly depleted from tears in human dry eye. The most novel and interesting finding was that CLU binds selectively to the damaged ocular surface. In this position, CLU protects against epithelial cell death and barrier proteolysis, and dampens the autoimmune response, while the apical epithelial cell layer is renewed. When present at high enough concentration, CLU also blocks staining by vital dyes used clinically to diagnose dry eye. None of the current therapeutics have this combination of properties to “protect, seal, and heal”. Future work will be directed towards human clinical trials to investigate the therapeutic promise of CLU.

## 1. Ocular Surface Disease

The wet mucosal ocular surface comprises the squamous multilayered epithelia of the cornea and conjunctiva, and the overlying tear film [1]. The cells are continually renewed in a process whereby daughter cells generated by division at the basal lamina are displaced upward in the cell layers. As they approach the surface, cells increasingly flatten. Cells at the most apical layer display an intricate array of membrane folds called microplicae, from which membrane-associated mucins project into the overlying tear film. Carbohydrate groups branching from the mucin protein backbones bind multiple oligomers of the galectin LGALS3 to form a highly organized glycocalyx which creates a transcellular barrier [2,3]. Tight junctions seal space between adjacent apical cells to create a paracellular barrier [3].

As the apical mucosal cells mature further, the microplicae flatten and membrane-associated mucin expression declines [4]. Metabolism also declines as the cells enter a specialized cell death pathway coincident with desquamation [5]. In humans, complete turnover of the ocular surface epithelia occurs in this way over five to seven days [6,7].

Ocular surface disease disrupts the normal progression, maturation and turnover of the ocular surface epithelia, leading to glycocalyx and cell damage, loss of apical cells by apoptosis, and barrier disruption. This pathology is caused by a variety of stress and disease conditions, including meibomian gland disease (MGD), rosacea, blepharitis, allergies, toxicities from glaucoma medication preservatives, as well as ultraviolet light and chemical/thermal burns [1]. Ocular surface disease is also commonly described in Sjögren’s syndrome, mucous membrane pemphigoid, graft-vs-host disease and a myriad of other autoimmune disorders [8,9,10].

Ocular surface disease initiated by tear dysfunction can be categorized under a syndrome known as dry eye, a common affliction associated with aging that affects 5% to 34% of all people globally [11]. Dry eye was defined by the 2017 Dry Eye Workshop (DEWS II) as “a multifactorial disease of the ocular surface characterized by a loss of homeostasis of the tear film, and accompanied by ocular symptoms, in which tear film instability and hyperosmolarity, ocular surface inflammation and damage, and neurosensory abnormalities have etiologic roles” [12]. Loss of tear film homeostasis has a variety of causes. Evaporative dry eye, the most prevalent subtype, is primarily due to MGD, which leads to lipid-poor tears that evaporate too quickly. Aqueous-deficient dry eye, the next most prevalent subtype, is caused by too little lacrimal gland fluid secretion. Most patients are mixed.

Ocular surface damage due to dry eye or other causes is typically evaluated in the clinic by staining with vital dyes such as fluorescein, rose bengal or lissamine green [13]. Considered a “sign” of disease, vital dye staining is one of the two outcome measures used in assessment of experimental therapeutics for dry eye. The other is symptoms, which may include blurry vision, discomfort or pain, redness and itching, and in severe cases, corneal scarring [14,15]. Each outcome measure is evaluated semi-quantitatively. Staining is scored using a grading system (e.g., [16]) and symptoms are scored using a set of multiple choice questions (e.g., [17,18]).

## 2. MMP9 Is Causal in Ocular Surface Disease

A team led by Stephen Pflugfelder at the University of Miami took up the problem of ocular surface disease mechanisms in the late 1990s, at a time when little was known. Pflugfelder had observed that patients with ocular rosacea have a significantly elevated concentration of the proinflammatory cytokine IL1A in their tear fluid [19]. He was aware that interleukin-1 isoforms increase the production of matrix metalloproteinases (MMP) [20]. Enzymes of the MMP family catalyze degradation of extracellular matrix (ECM) proteins, as well as other proteins associated with pathological tissue damage, including cytokines and cell adhesion molecules (reviewed in [21,22]). It seemed likely that MMPs could be key mediators of ocular surface damage in disease.

The MMP family includes 24 independent gene products in humans. Enzymatic activity of MMPs is dependent on the presence of zinc in the catalytic domain. Because of their potential for destruction, MMP activity is tightly controlled at both transcriptional [20] and post-transcriptional levels [23]. All MMPs can be inhibited by endogenous tissue inhibitors of metalloproteinases (TIMPs) [24].

**Figure 1:**
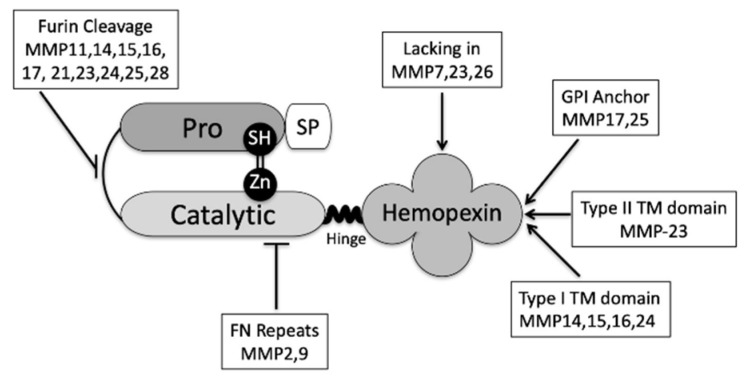
Metalloproteinase (MMP) domain structure. The typical MMP has a structure like MMP1, comprising an N-terminal propeptide of about 80 amino acids, a catalytic domain of about 170 amino acids, a hinge domain of variable length, and a hemopexin domain of about 200 amino acids. MMP7, MMP23 and MMP26 are exceptions as they lack the linker and the hemopexin domains. Ten of the MMPs share a furin-like proprotein convertase motif located between the propeptide and catalytic domains. MMP2 and MMP9 have three type II fibronectin repeats in the catalytic domain. MMP14, MMP15, MMP16, and MMP24 are not secreted, but instead remain tethered to the plasma membrane via a type I transmembrane domain with a short cytoplasmic tail. MMP23 contains a type II transmembrane domain. MMP17 and MMP25 are secreted, but then attach to the plasma membrane by means of a glycosylphosphatidylinositol (GPI) anchor added to the C-terminus by posttranslational modification. Adapted from [32].

MMPs were traditionally described as secreted proenzymes or “zymogens”, which are activated in the extracellular environment. However, several membrane-tethered forms are now known, and these (as well as some other family members) are activated intracellularly. MMPs share a set of structural domains that confer their specific functions and overlapping substrate specificities (reviewed in [21,25,26,27,28,29,30,31,32]). These are depicted graphically in Figure 1.

Pflugfelder was familiar with work from our lab demonstrating that the corneal epithelium can produce MMPs [33,34,35]. We had shown, using normal and pathological animal models, combined with analysis of pathological human tissues that (1) expression of MMP9 is induced in ocular surface epithelia during normal wound repair, (2) an elevated level of MMP9 is produced in nonhealing wounds associated with basement membrane defects, and (3) broad-spectrum MMP inhibitors reduce these defects [36,37,38]. Hypothesizing that MMPs might be similarly involved in ocular surface disease, Pflugfelder and his team demonstrated elevated MMP9 levels in the tears of human patients with ocular rosacea [39,40] and dry eye [41] (reviewed in [42]). Later they showed that patients with dysfunctional tear syndrome had elevated MMP9 enzymatic activity in their tears, and the amount of activity correlated with disease severity. In addition, MMP9 expression was higher in conjunctiva from diseased patients, as assayed by impression cytology [43].

Pflugfelder worked with Michael Stern from Allergan to develop one of the first mouse models for dry eye [44,45]. Aqueous tear production was inhibited by cholinergic blockade. In the initial report, this was done by application of transdermal scopolamine patches [44]; in later versions, scopolamine was administered by intramuscular injection [45]. Desiccating stress at the ocular surface was amplified by placing mice in front of a fan to provide continuous airflow. Ocular surface damage was assessed after five days by measurement of fluorescein or Oregon green dextran staining. Importantly, mice subjected to desiccating stress exhibited stimulated expression and production of IL1B and MMP9, as in people [45].

With availability of a mouse model, it became possible to investigate causality of MMP9 in ocular surface disease. MMP-deficient “knockout” mouse strains engineered using traditional gene-targeting technology have provided important information about MMP roles in biology and pathology (reviewed in [31]). A general observation is that loss of individual MMPs has little effect on embryonic development. The exception is MMP14, deficiency of which results in bone defects. The lack of developmental phenotypes for MMP9 knockout mice has been explained by the fact that MMP substrates overlap extensively, and multiple MMPs likely assume the same role in developmental processes.

A deficiency of individual MMPs also has little effect on tissue and organ homeostasis in the adult. This is not particularly surprising considering that MMPs are generally not expressed in normal, non-remodeling tissues. However, knockout mouse phenotypes become clearly apparent when tissues are stressed and homeostasis is perturbed, for example during wound healing or infection. Such challenges result in the induction of specific MMP expression. Comparison of results in wild-type mice with knockout mice in these situations has provided strong evidence for the causal involvement of MMPs in the process.

Teams led by Zena Werb at the University of California San Francisco and Robert Senior at Washington University, independently set out to generate *Mmp9* knockout mice at approximately the same time. Ultimately the two decided to work together, and they co-authored the first paper on the *Mmp9* knockout mouse phenotype [46]. Robert Senior approached us early on about using the knockout mouse to investigate the role of MMP9 in repair of the ocular surface epithelia [47]. While we were conducting these studies, the Pflugfelder lab contacted us about a collaboration on dry eye and Dr. Senior generously agreed to provide the mice.

For these early studies, the *Mmp9* knockout allele was on the mouse strain 129SvEv/CD-1 mixed genetic background, and we used matched wild-type littermates as controls. As expected, ocular surface staining with vital dyes increased in wild-type littermate mice subjected to desiccating stress, and this was accompanied by an increase in the concentration of MMP9 protein in tear fluid. In striking contrast, ocular surface staining did not change in knockout mice. In a “rescue” experiment, topical administration of activated MMP9 protein to the eyes of *Mmp9* knockout mice significantly increased ocular surface staining in mice subjected to desiccating stress. Compared to *Mmp9* knockout mice, wild-type mice subjected to desiccating stress showed greater loss of differentiated apical epithelial cells that expressed the tight junction protein OCLN (occludin). This was accompanied by an increase in a truncated form of OCLN in the corneal epithelia of wild-type, but not knockout mice. These findings were replicated in cultured human corneal epithelial cells treated with activated MMP9 protein. The results indicate that increased MMP9 enzymatic activity on the ocular surface in response to desiccating stress is causal in ocular surface disease, and suggest that this is due, at least in part, to proteolysis of OCLN protein located in epithelial tight junctions [48].

## 3. The Molecular Chaperone Clusterin Is a MMP9 Inhibitor

With the goal to identify other proteins interacting with MMP9 that might contribute to its role in ocular surface disease, we conducted a yeast two-hybrid screen [49]. MMP9 was used as bait to screen a cDNA library prepared from human corneal RNA. CLU (clusterin) emerged as an interacting protein. CLU was found to bind very strongly to a truncated form of MMP9 lacking the pro-domain, with an affinity constant of 2.63 nmol/L.

CLU is a homeostatic, evolutionarily-conserved, secreted glycoprotein, expressed prominently by epithelia at fluid-tissue interfaces and found in all bodily fluids [50,51]. Its primary role is to protect cells and tissues against damaging stress [52,53]. The first reports on CLU appeared in 1983 [54,55] and there are now thousands of publications.

CLU was discovered in degenerating tissue systems by several different labs independently. Based on this association, it was proposed that the protein promotes apoptotic cell death. However, it was subsequently found to do the reverse: induction of CLU expression protects against apoptosis (reviewed in [56]).

In 1999, the lab of coauthor Mark Wilson at the University of Wollongong in New South Wales, Australia reported that secreted CLU functions as a molecular chaperone, binding preferentially to misfolded proteins to stabilize their structure and inhibit their aggregation [57]. Examples of molecular chaperones are the heat-shock proteins (e.g., HSP70, HSP90), first characterized in fruit flies [58]. The novelty of Wilson’s discovery was that CLU was the first molecular chaperone identified that was located *outside* of the cell. Like other molecular chaperones, CLU binds selectively to exposed regions of hydrophobicity on misfolded or denatured proteins, solubilizing them to prevent their aggregation and precipitation [59,60]. Immunodepletion of CLU from blood renders plasma proteins vulnerable to stress-induced aggregation [61]. CLU knockout mice spontaneously develop protein deposits in the kidney, and glomerular neuropathy, with aging [62]. Moreover, increased tissue damage is seen in CLU knockout mice following application of acute stress [63] or in various disease models, including a myosin-induced autoimmune myocarditis model [64] and postischemic brain injury [65]. Conversely, damage due to ischemic brain injury is reduced by CLU over-expression [65].

Several mechanisms have been described for how CLU inhibits cell death; in one, CLU binding to cell surface low-density lipoprotein receptors such as LRP2 (megalin) [66], LPR8, or VLDLR activates signaling pathways promoting cell survival [67].

Both reduced and elevated levels of secreted CLU are associated with disease states. The former likely represents dysfunction, and the latter a compensatory stress response [60]. CLU is found in molecular aggregates associated with diseases of protein and lipid deposition, such as Alzheimer’s disease, and atherosclerosis [68]. Secreted CLU is also found in protein aggregates in several eye diseases, including corneal dystrophies, pseudoexfoliation glaucoma and macular degeneration (reviewed in [56]). The presence of CLU in insoluble aggregates is thought to represent a local overloading of CLU’s capacity to inhibit protein aggregation [56,68]. However, if CLU can attain a sufficient concentration threshold, it potently inhibits protein aggregate formation and provides substantial cytoprotection [52].

**Figure 2:**
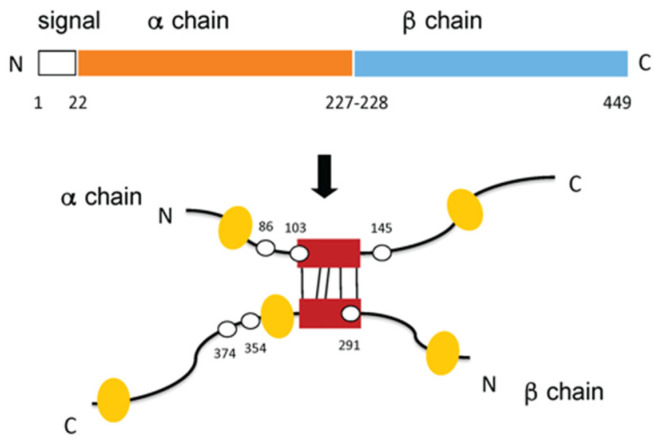
Primary structure of secreted clusterin. Secreted CLU is composed of two polypeptide chains connected by five disulfide bonds, derived from an intracellular precursor. In the first processing step, the 22-mer secretory signal peptide is cleaved from the 449-amino acid precursor. Subsequently the chain is cleaved again between Arg227-Ser228 to generate an *a*-chain and a *b*-chain. These are assembled in antiparallel fashion to generate a heterodimeric molecule in which the cysteine-rich centers (red boxes) are linked by five disulfide bridges (black lines). The mature protein is 17–27% N-linked carbohydrate by mass. The three sites for N-linked glycosylation on each chain are clustered around a central region of ordered structure stabilized by the disulfide bonds forming a core that is hydrophilic due to the glycosyl groups. The six sites for N-linked glycosylation are indicated (white ellipses). Five predicted amphipathic *a*-helices (yellow ovals) in the otherwise disordered arms may be important for binding to hydrophobic regions exposed on misfolded proteins and for insertion into lipid structures. Amino acid numbering for the N- and C-termini, the cleavage sites, and the six sites for N-linked glycosylation (white ovals) are indicated. (From (56) with permission from the publisher, order number 501613529).

CLU has structural features shown in Figure 2. It is synthesized on ribosomes associated with the cytosolic face of the endoplasmic reticulum (ER) and is co-translationally translocated into the ER lumen, where a 22-mer signal peptide is proteolytically removed. Glycosylation is begun in the ER lumen and more complex sugars are attached in the Golgi. Prior to its secretion from the cell, CLU is also folded, disulfide-bonded, and proteolytically-processed inside the ER/Golgi. CLU found within the epithelia at fluid-tissue boundaries is in transit between synthesis and secretion (or release following cell death). Historical literature claims that CLU has intracellular isoforms (nuclear and cytoplasmic) produced by alternative splicing, however, this line of research has not been substantiated over the years. The current consensus is that NCBI Genbank transcript NM_001831.3 is translated to produce the vast majority of CLU protein and that non-secreted CLU isoforms are rare (reviewed in [69]).

What might be the biologic implications of MMP9-CLU interaction? We proposed two possibilities: (1) CLU was a substrate for MMP9 enzymatic activity, or (2) CLU modulated MMP9 activity, possibly as an inhibitor. The latter hypothesis proved to be true. CLU was found to inhibit the enzymatic activity of MMP9, comparing quite favorably to the synthetic small molecule inhibitor SB-3CT. CLU also was found to inhibit enzymatic activity of MMP2 and MMP3, and to a lesser extent, MMP7. These results are consistent with those of an earlier study in which CLU was discovered to be a binding protein and enzymatic inhibitor of MMP25 [70].

The biochemical mechanism of CLU inhibition of MMP activity remains to be investigated. Interestingly, another extracellular chaperone, A2M (alpha-2-macroglobulin) [71], is also a broad-spectrum proteinase inhibitor, with MMP inhibitory activity [72]. It is intriguing to suggest the idea that proteinase inhibition is part of an anti-inflammatory suite of activities shared by extracellular chaperones.

## 4. Clusterin Protects the Ocular Surface in Mouse Dry Eye

In 1996, a group led by Shigeru Kinoshita reported that CLU mRNA is the most abundant transcript in the human corneal epithelium. The protein product was found to accumulate in the apical cell layers adjacent to the tear film [73,74]. CLU was later identified in human tears [75,76]. CLU expression in the corneal epithelium was greatly reduced in severe ocular surface disease with dry eye [77,78]. This suggested the hypothesis that CLU protects the mucosal ocular surface epithelia against desiccating stress and that a reduction in CLU leaves the ocular surface vulnerable to damage.

We found immunoreactive CLU protein to be localized within the subapical ocular surface epithelial cells of mice, much as described in the Kinoshita study of humans [77]. When mice were subjected to desiccating stress, MMP9 immunostaining in the epithelium was increased as expected. In contrast CLU immunostaining was substantially diminished. Again, the finding with CLU was much like the Kinoshita findings in humans [77,78]. The results suggested that one way CLU protects the ocular surface is by inhibiting MMP9.

To test our hypothesis about the role of CLU in ocular surface disease, CLU levels were perturbed while mice were subjected to desiccating stress by (1) supplementing via topical drops in wild-type mice, and (2) using CLU knockout mice. Ocular surface damage was assessed by vital dye staining [79]. In wild-type mice, drops containing human plasma CLU, recombinant human CLU, recombinant mouse CLU, or a bovine albumin control, were delivered topically, four times/day in a dose-response experiment. In all cases, CLU at a concentration of 1–3 ug/mL was found to prevent ocular surface damage or ameliorate pre-existing damage, bringing vital dye staining to baseline. CLU concentration in normal mouse tears was somewhat above the effective supplementation threshold (~5 ug/mL), but dropped by about one third in mice subjected to desiccating stress. In heterozygous CLU knockout mice deficient in tear CLU, the ocular surface was found to be more vulnerable to desiccating stress.

In our final set of experiments, we investigated the requirement for continual delivery of CLU [79]. We found that a single topical application of CLU at 3–6 ug/mL—a dose somewhat higher than the amount needed for protection (1–3 ug/mL)—prevented fluorescein vital dye uptake immediately. A single topical CLU application at this higher dose sealed the ocular surface against vital dye staining for at least two hours.

We conclude that natural tear CLU protects the ocular surface in mouse dry eye, but that the amount in tears is limiting and may become insufficient to prevent damage to ocular surface cells and proteins. Topical CLU supplementation to restore the physiologic level, both protects and promotes healing [79].

## 5. Clusterin in Human Tears Is Reduced in Dry Eye

To determine whether CLU might also be reduced in the tears of human patients with dry eye, we performed a clinical study [80]. A positive correlation was observed between results of the Schirmer strip test—a measure of tear production—and tear CLU concentration. In other words, the CLU concentration was lower in aqueous-deficient dry eye, consistent with our findings in mice.

In the most comprehensive mass-spectrometry-generated list, 1543 tear proteins were identified, most present at very low levels [76]. However, a small number of highly abundant, and abundant proteins comprise more than 90% of the total tear protein by weight, including LYZ (lysozyme), LTF (lactoferrin), LCN1 (tear lipocalin) and LACRT (lacritin) [81]. The concentration of CLU in normal human tears was measured as ~30 ug/mL in our clinical study [80]. This is ~10 times lower than the abundant tear protein LACRT, and ~50 times lower than the very abundant tear protein LYZ. As evaluated by SDS gel electrophoresis, clinical tear CLU was similar biochemically to CLU obtained from both a nonclinical tear sample and from blood.

These findings are consistent with the idea of a limited CLU concentration in tears, which can be easily overwhelmed.

## 6. Clusterin Binds Selectively to the Damaged Ocular Surface to Protect and Seal

In our next set of experiments, we investigated CLU mechanisms of action. First we tested CLU’s capacity to protect the cells and proteins of the barrier against physical damage [79]. Ocular surface damage was evaluated by (1) terminal deoxynucleotidyl transferase dUTP nick end mabeling (TUNEL) assay on corneal tissue sections, to quantify cells undergoing apoptosis, and (2) by gel electrophoresis of proteins extracted from the ocular surface epithelia, to evaluate proteolysis of barrier proteins. CLU treatment of the ocular surface reduced apoptosis of ocular surface epithelial cells due to desiccating stress. CLU treatment also reduced proteolytic damage to two different ocular surface barrier proteins: OCLN, a component of the paracellular barrier, and LGALS3 (galectin-3), a component of the transcellular barrier. The former is consistent with CLU’s activation of antiapoptotic pathways and the latter is consistent with its action as a MMP9 inhibitor. We further found that treatment of ocular surface epithelial cells in culture with CLU inhibits MMP9 expression in response to the inflammatory cytokine TNFA (Transforming Growth Factor Alpha). Thus, CLU also inhibits MMP9 proteolytic activity by preventing MMP9 synthesis.

**Figure 3:**
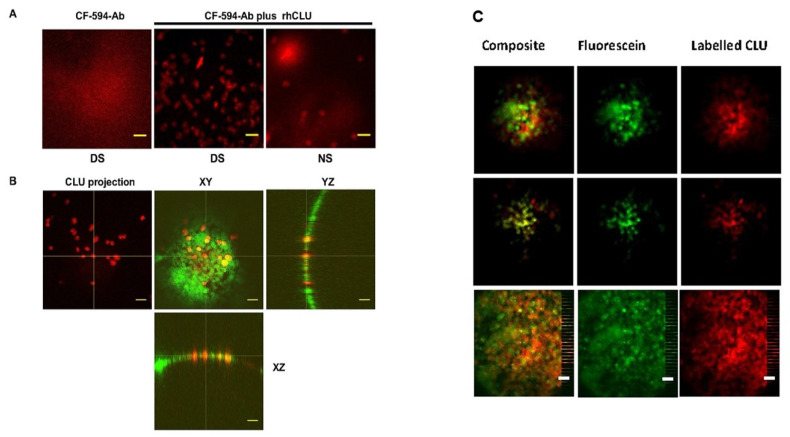
CLU binding to the ocular surface. (**A**) The standard desiccating stress protocol was applied for five days to create ocular surface damage. Nonstressed (NS) mice housed under normal ambient conditions were included for comparison. Eyes were treated with CF-594-anti-His antibody that binds to the His tag of recombinant human CLU (rhCLU), or with a complex of the antibody-rhCLU for 15 min, followed by confocal imaging of central cornea. (**B**) An eye subjected to DS was treated with a complex of the antibody-rhCLU (red) as in (**A**), as well as a fluorescent membrane tracer DiO (green). Images were taken at 20× magnification. In the left panel only CLU was projected. The right three panels show one Z-section plane with cross-sections oriented to the XY, YZ, and XZ axes, generated using Image J software. Yellow indicates regions of co-localization of the red and green signal. (**C**) On the morning of the 6th day after the DS protocol was begun, labeled CLU was mixed with clinical fluorescein dye Fluoresoft^®^-0.35% (Holles Laboratory, Cohasset, MA; 2 μL CLU + 1 μL fluorescein) and then 2 μL of the resulting mixture instilled to the OcS for 15 min. Eyes were then removed and the OCS was imaged by confocal microscopy with simultaneous two-color excitation (10× magnification). Fluorescein and CLU were distinguished by the color of fluorescence emission (far-red for CLU, green for fluorescein, please note difference from Fig 1B, where green is DiQ). The three rows represent different focal planes on a single cornea. 10× magnification. Scale bar = 100 μm; (**A**,**B**) from (79) CC-BY; used with permission from the publisher.

Next, we investigated the mechanism of sealing [79]. Vital dye staining in dry eye occurs in a characteristic punctate pattern representing transcellular uptake by individual cells. These cells are located primarily in the apical layer, but because of damage to tight junction proteins, dye also penetrates the paracellular barrier and is taken up by some cells in lower layers [82,83]. We showed that CLU binding is selective for the stressed ocular surface and the binding pattern is punctate, like staining with vital dyes [79]. Confocal Z-sectioning revealed that CLU is localized to the plasma membrane, or within individual cells of the apical cell layer (Figure 3A,B).

In a follow-up experiment, we attempted to understand the relationship between CLU binding and fluorescein uptake. Since CLU binds only to the stressed ocular surface, we used stressed eyes only for this study. We found that CLU and fluorescein overlap substantially, in the same punctate pattern (Figure 3C). These results (previously unpublished) suggest that CLU is selectively bound by the same cells that take up fluorescein.

How does CLU recognize ocular surface damage? CLU would be expected to bind to any misfolded or denatured client protein in its role as a molecular chaperone [57,84]. CLU also binds lipids [85], with selectivity for oxidized lipids [86] that would likely be more prevalent on the surface of damaged cells. In our final set of experiments, we showed that CLU binds to the glycocalyx component LGALS3 [79]. We suggest this may be one binding site at the damaged ocular surface, perhaps made available due to proteolytic activity. More recently, Wilson and colleagues demonstrated that CLU also binds selectively to histones exposed on cells in the late stages of apoptosis [87].

**Figure 4:**
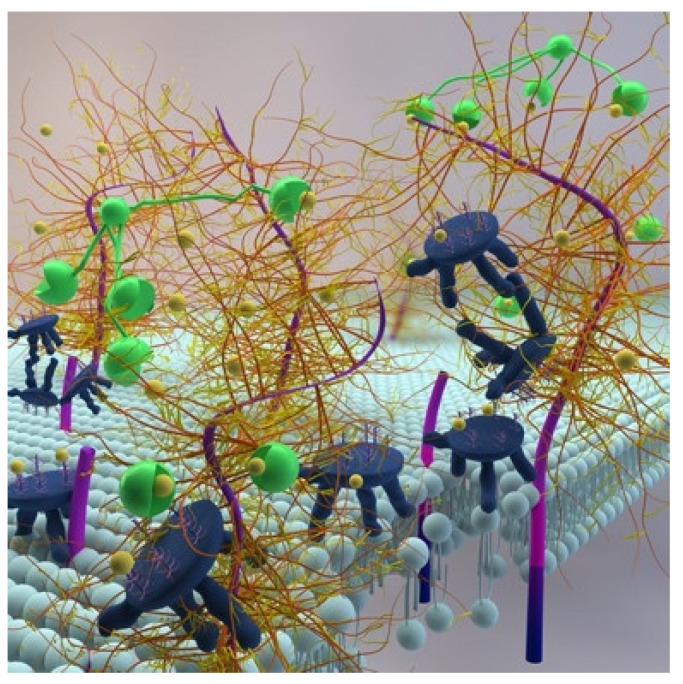
Conceptual model of CLU protection and sealing of the damaged ocular surface. Damage to the mucosal glycocalyx due to proteolysis and denaturation and damage to the plasma membrane due to oxidation is sealed by CLU via solubilization and lipid bilayer intercalation. The blue “stools” represent CLU molecules. The gold “brushes” represent membrane-associated mucins. The green “mouths” represent LGALS3 multimers. The light blue “hairpins” represent the lipid bilayer. (updated from (56)).

Based on our results, we propose a model whereby CLU binds selectively to the damaged ocular surface. Thus situated, CLU protects against further damage due to apoptosis, protein denaturation and MMP proteolysis, thus supporting healing. When present at sufficient concentration, CLU effectively seals breaches in the ocular surface barrier, acting as a “bandage” to prevent vital dye staining [79]. Figure 4 depicts our “protect, seal and heal” conceptual model.

## 7. Clusterin Dampens the Autoimmune Response

Current thought holds that dry eye progresses via an amplifying process of inflammation that involves reciprocal interactions between the tear film and immune cells, and the ocular surface epithelia. Acute desiccating stress due to tear dysfunction activates signaling pathways in resident immune cells and epithelia, triggering production of inflammatory mediators and MMPs. Inflammatory cells are recruited, local dendritic cells undergo maturation, and barrier disruption develops by protease-mediated lysis of glycocalyx and epithelial tight junctions. Epithelial cell damage causes exposure of autoantigens. The result is an autoimmune-like adaptive T cell-mediated response. Conjunctival goblet cell dysfunction and death are promoted by IFNG (Interferon Gamma). These changes further destabilize the tear film, creating a “vicious cycle of inflammation”, viewed as a core driver of the disease (reviewed in [88,89]).

CLU was characterized early on as an exchangeable apolipoprotein in the blood (APOJ), which associates with circulating high-density lipoprotein having anti-inflammatory activity [90,91]. In addition, CLU was identified in immune deposits as part of the complement membrane attack complex and shown to inhibit complement-mediated cytolysis in vitro by binding to subunit SC5b-9 (reviewed in [56]). Elevated levels of CLU observed during inflammation could reflect a compensatory protective mechanism. Evidence continues to accumulate for the anti-inflammatory properties of CLU in different systems (e.g., [92,93]). 

In a recent study by Wilson and colleagues [87], selective CLU binding to apoptotic cells was shown to potentiate their phagocytosis by macrophages. In a model of apoptotic cell-induced autoimmunity, and relative to control mice, CLU knockout mice developed symptoms of autoimmunity, including the generation of anti-dsDNA antibodies, deposition of immunoglobulins and complement components within kidneys, and splenomegaly. These results provide strong evidence that CLU binding to damaged cells dampens the autoimmune response. 

Based on these findings, we suggest that CLU could act to ramp down the inflammatory vicious cycle in dry eye. Consistent with this hypothesis, is our observation in a cell culture model, discussed above, that CLU inhibits expression of MMP9 in response to the inflammatory cytokine TNFA, [79]. However, whether this occurs in a dry eye model in vivo remains to be tested directly. 

## 8. Therapeutic Potential of Clusterin

There is a great need for better strategies to manage dry eye. Treatment with over-the-counter artificial tears and drops can provide some symptom relief. However, these therapies are soothing, but not healing. Moreover, active ingredients (e.g., hyaluronic acid; polyethylene glycol) wash out of the eye in minutes, and, while present, can cause blurry vision [94].

Small molecule analogues of the natural anti-inflammatory hormone cortisol are used off-label to treat dry eye. However, steroids can raise intraocular pressure, which can lead to glaucomatous optic neuropathy [95]. They must be used carefully. Two drugs are currently approved specifically for the treatment of dry eye by the US Food and Drug Administration (FDA): Cyclosporine A (Restasis, Allergan, FDA-approved 2002) and Lifitegrast (Xiidra, Shire, FDA-approved 2016). They are also anti-inflammatory small molecules. Both target T-cell function [96]. Both have undesirable side-effects such as burning sensation and bad taste. They do not help all patients, being more effective for aqueous-deficient dry eye. Even in patients that are helped, improvement can take many months [94]. Moreover, these drugs are not a cure and must be used indefinitely.

The lack of effective FDA-approved pharmaceuticals for all dry eye subtypes has led ophthalmologists to seek alternatives. Many proteins present in tears are anti-inflammatory, cytoprotective and promote healing, and most tear proteins are also present in blood [81]. For these reasons, there is growing interest in the use of “autologous serum tears” (AST), to treat dry eye.

AST are manufactured from the serum of the individual to be treated, thus avoiding any complications due to immunogenicity or infection. Recent short-term comparative and randomized studies [97,98,99,100] provide evidence that AST may be effective for dry eye [100,101], Sjögren’s syndrome [102] and graft-vs-host disease [103,104,105]. Unfortunately, the heterogeneity of AST makes it very difficult to conduct long-term, well-controlled trials [100]. Of concern, patients with systemic autoimmune diseases may have altered composition of their serum [106,107], thus, their AST may be ineffective [106,108], and even toxic [109,110]. In addition, this approach may not be the most accessible or cost-effective.

A more ideal approach would be to identify key active components of AST, and then manufacture them in a pure form using recombinant DNA technology. This would eliminate the need for continuous phlebotomies or heterogenous preparation methods, and such a drug would not require refrigeration [111]. The concentration of CLU in human serum is ~100 ug/mL, which is three times the concentration that we measured in human tears [80]. Thus, CLU may well be a key component of AST.

Recombinant protein drugs are part of a broader class called “biologics”, that represent the largest group of new products under development by the biopharmaceutical industry [112]. Biologics address the high attrition rate of small molecules in preclinical and clinical trials, ascribed to toxicity, insufficient efficacy or inadequate selectivity [113]. Biologics are held by the FDA to rigorous safety standards, and their defined nature makes it possible to test their efficacy in well-controlled clinical trials.

The Wilson lab has described a rapid and efficient method to produce structurally and functionally-validated recombinant human CLU at high yields [114]. This is an important first step towards manufacturing CLU as a drug. CLU is highly conserved across evolution, exhibiting 80% sequence similarity between humans and mice. Importantly, recombinant human CLU and recombinant mouse CLU worked equally well to prevent dry eye in our mouse model [79]. Thus, safety testing in mammalian species is unlikely to pose a problem. The process must still be adapted for compliance with Good Manufacturing Practices (GMP).

A recent publication raises the intriguing new idea that dry eye is a disease of protein aggregation, placing it in the same class as Alzheimer’s, Parkinson’s disease and related synucleinopathies, as well as pseudoexfoliation glaucoma [115]. Supplementation with CLU to enhance molecular chaperone activity has been proposed as a treatment for some of these diseases [116,117,118,119]. CLU was recently shown to protect rod photoreceptors in a rat model of retinitis pigmentosa [120], highlighting another disease that might be treated with a CLU biologic. It will be exciting to learn whether CLU can be effective in treating ocular surface disease in dry eye, as well as other disorders of protein aggregation.

## 9. Conclusions

Evidence is presented herein supporting potential of the natural homeostatic glycoprotein CLU as a novel therapeutic for the treatment of dry eye. Future work will be directed towards human clinical trials to investigate the therapeutic promise predicted by these findings.

## 10. Patents

M.E.F. and S.J. are named as coinventors on US patent number 9241974 entitled “Clusterin Pharmaceuticals and Treatment Methods Using the Same” granted to the University of Southern California.

## Data Availability

All original data is included in this paper.

## Abbreviations

Hugo nomenclature used for genes and their products

AST	autologous serum tears
DEWS II	Dry Eye Workshop
ECM	extracellular matrix
ER	endoplasmic reticulum
FDA	Food and Drug Administration
GMP	Good Manufacturing Practices
MMP	matrix metalloproteinase
MGD	Meibomian gland disease
TIMP	tissue inhibitors of metalloproteinases
